# A Scoping Review of Thermal and Nonthermal Technologies for *Listeria monocytogenes* Inactivation in Ready‐to‐Eat Meats

**DOI:** 10.1111/1541-4337.70506

**Published:** 2026-05-13

**Authors:** Mustafa Guzel, Ilhami Okur

**Affiliations:** ^1^ Department of Food Engineering Hitit University Çorum Türkiye; ^2^ Department of Food Science and Technology University of Nebraska‐Lincoln Lincoln Nebraska USA

**Keywords:** emerging technologies, *Listeria monocytogenes*, microbial inactivation, ready‐to‐eat meat, thermal lethality

## Abstract

*Listeria monocytogenes* continues to present a significant food safety challenge in ready‐to‐eat (RTE) products, despite existing regulatory standards and antimicrobial intervention strategies. To manage the risk of *L. monocytogenes* in RTE meats, food producers and researchers have explored a range of intervention strategies, broadly categorized into thermal treatments and nonthermal technologies. This scoping review aimed to map and synthesize the available evidence on the effects of these technologies against *L. monocytogenes* in RTE meats. Three electronic bibliographic databases (Scopus, PubMed, and Web of Science Core Collection) were searched using terms that reflected three concepts: RTE meat, *L. monocytogenes*, and microbial inactivation. Of 1111 records initially retrieved, 74 studies met the inclusion criteria. High‐pressure processing (HPP) was the most frequently investigated technique for inactivating *L. monocytogenes* in RTE meats (29/74, 39.2%), followed by thermal treatment (18/74, 24.3%). Thermal processes and ionizing radiation generally produced the most consistent and substantial reductions across the reviewed studies. However, several nonthermal approaches, including HPP, cold plasma, pulsed light, and ultraviolet (UV) light, showed more variable performance, depending strongly on treatment intensity, product characteristics, and study conditions. These findings indicate that intervention performance in RTE meats should be interpreted in relation to process parameters, matrix effects, and posttreatment behavior during storage rather than by reduction magnitude alone. Overall, this review provides an RTE meat‐focused synthesis of the current evidence base and identifies methodological priorities for future studies evaluating thermal and nonthermal control strategies for *L. monocytogenes*.

## 1 Introduction

Ready‐to‐eat (RTE) meat products are defined as processed or prepared meats intended for consumption without the need for further cooking or microbial kill steps. In European Union regulations, an RTE food is defined as “food intended by the producer or manufacturer for direct human consumption without the need for cooking or other processing effective to eliminate or reduce to acceptable levels microorganisms of concern” (EFSA Panel on Biological Hazards (BIOHAZ) 2018). This category encompasses a wide variety of foods, including cooked or cured luncheon meats, deli slices from pork, poultry, or beef, sausages and hot dogs, pâtés, and fermented or dried meat products (Stessl et al. 2022). Since RTE products receive no further cooking by the end user, RTE meat safety depends on the effectiveness of industrial processing controls to eliminate or reduce pathogens (WHO 2022). RTE meat processing relies on multiple hurdle preservation techniques. Following an initial lethal heat treatment, interventions such as salting or curing (to reduce water activity), smoking, drying, and fermentation or acidification are applied to inhibit bacterial growth (Molina et al. 2024). Chemical preservatives (e.g., lactate salts or nitrite) are also commonly incorporated to suppress pathogens and spoilage microbes. These measures are essential to extend shelf life and ensure the safety of RTE meats (Stessl et al. 2022). In this context, the public health significance of RTE meats is shaped not only by the effectiveness of the initial processing step, but also by the possibility of postprocess contamination, persistence in the processing environment, and growth during storage and distribution. EFSA has emphasized that RTE foods are a broad and diverse category, and that the ability of a given product to support *L. monocytogenes* growth depends on product characteristics, packaging conditions, storage temperature, and shelf life (EFSA Panel on Biological Hazards (BIOHAZ) 2018).

*L. monocytogenes* contamination in RTE meats poses a persistent public health challenge due to this pathogen's ubiquitous presence and the severe disease it can cause. Although human listeriosis is relatively rare (<1 case per 100,000 population), it primarily affects immunocompromised individuals such as older adults and pregnant women and carries a high fatality rate of approximately 15%–30% in these vulnerable groups (Choi et al. 2018; EFSA Panel on Biological Hazards (BIOHAZ) 2018). In recent years, surveillance data indicate an upward trend in listeriosis cases in many regions. For example, nearly 2993 confirmed cases were reported in EU countries in 2023, the highest annual total on record, with the highest incidence observed among adults aged 64 and older (ECDC 2025). In North America, *L. monocytogenes* has also caused significant public health problems, including a 2008 Canadian deli meat outbreak that resulted in 57 cases and 24 deaths (Vidovic et al. 2024). A recent QMRA model estimated that RTE deli meats accounted for the majority of sporadic listeriosis cases in the United States, with over 90% of cases (Sampedro et al. 2022). *L. monocytogenes* is challenging to control in RTE food settings because it tolerates stress conditions and can persist in food processing environments. *L. monocytogenes* can survive and even grow at refrigeration temperatures, withstand high salt or acidic conditions, and form biofilms on equipment and surfaces, which protect it from sanitizers (Mazaheri et al. 2021; Osek et al. 2022). Thus, even when RTE meats are produced under hygienic conditions, low‐level postprocess contamination is not uncommon. Surveys in Europe have found *L. monocytogenes* in roughly 3%–5% of assorted RTE food samples at retail (EFSA and ECDC 2022). Systematic reviews of meat products reported various contamination rates in RTE meats. For instance, approximately 3.2% of RTE meat samples in China and 11% in Brazil were positive in a recent meta‐analysis (Cavalcanti et al. 2022; Y. Liu et al. 2020). In the United States, FSIS testing of more than 158,000 RTE meat and poultry samples between 2005 and 2017 found that only 0.33%–0.36% were positive for *L. monocytogenes*. This reflects a dramatic reduction from prevalence levels of ∼4.5% reported in the 1990s to ∼0.2% by 2017, attributed to the implementation of HACCP systems in 1996 and the FSIS “*Listeria* Rule” in 2003 (Mamber et al. 2020). Because these products are widely consumed without further cooking, even infrequent contamination can lead to human listeriosis. Indeed, RTE meats have been identified as a leading source of listeriosis cases. Recent large‐scale outbreaks and recalls of ready‐to‐eat meat and poultry products linked to *L. monocytogenes* have prompted the U.S. Department of Agriculture's Food Safety and Inspection Service to strengthen regulatory oversight. These measures, implemented beginning in 2025, include expanded *Listeria* species testing, enhanced inspection protocols, and intensified facility‐level risk assessments (USDA‐FSIS 2024).

To manage the risk of *L. monocytogenes* in RTE meats, food producers and researchers have explored a range of intervention strategies, broadly categorized into thermal treatments and nonthermal technologies. Thermal interventions refer to the application of heat to inactivate pathogens. Almost all RTE meat products undergo a cooking or pasteurization step as part of their manufacture. For example, cooking hams and frankfurters to a core temperature sufficient to achieve a 5‐log reduction of *L. monocytogenes* is a common industry target (EFSA Panel on Biological Hazards (BIOHAZ) 2018). Such heat treatments serve as the primary “kill step” in most RTE meat processes. However, because *Listeria* can be introduced or can regrow after the cooking step, additional interventions are employed to further control the pathogen on finished products. Many RTE meat formulations include intrinsic hurdles that act without high heat. For instance, low pH and low water activity in fermented/dry sausages create conditions that are inhibitory or lethal to *L. monocytogenes* over time. The use of certain food‐grade additives provides another layer of protection. Organic acid salts and nitrite are commonly added to RTE meat products to suppress *Listeria* growth during refrigerated storage (EFSA Panel on Biological Hazards (BIOHAZ) 2018).

Beyond these built‐in measures, postprocess nonthermal technologies have been developed to inactivate *L. monocytogenes* on RTE foods without additional cooking. One important example is high‐pressure processing (HPP). This physical process can destroy bacterial cells by membrane disruption and enzyme inactivation, achieving significant lethality against *L. monocytogenes* without heat (EFSA Panel on Biological Hazards (BIOHAZ) 2018). Another example is cold plasma technology, which employs an ionized gas at near‐room temperature that generates highly reactive species capable of disrupting microbial cell membranes and DNA. Studies have reported that cold plasma can achieve significant *L. monocytogenes* inactivation on RTE meats, and its efficacy can be further enhanced by combining with chilled storage posttreatment (Lis et al. 2018). UV‐C is also a promising treatment method. In UV‐C, short‐wavelength ultraviolet (UV) light (around 254 nm) is employed to inactivate microorganisms by causing DNA damage. UV‐C surface irradiation has been shown to reduce *L. monocytogenes* on food products (Lee et al. 2025).

Several comprehensive reviews and assessments have addressed *L. monocytogenes* in the food supply; however, the available literature remains dispersed across intervention classes, product categories, and regulatory contexts, which makes cross‐technology interpretation difficult within a single RTE meat framework. Most prior reviews have either examined the occurrence and epidemiology of *L. monocytogenes* or specific facets of control rather than surveying all intervention approaches (Belias et al. 2024; Cavalcanti et al. 2022; Churchill et al. 2019; Forauer et al. 2021; Y. Liu et al. 2020). In addition, the regulatory interpretation of *L. monocytogenes* in RTE foods is not uniform across jurisdictions. In the European framework, microbiological criteria are linked to both product category and the ability of the food to support growth during shelf life, whereas the USA applies a more hazard‐focused zero‐tolerance approach for detection in RTE foods (EFSA Panel on Biological Hazards (BIOHAZ) 2018; WHO 2022; Stessl et al. 2022). FAO/WHO has also noted that caution is warranted when classifying foods strictly as RTE or non‐RTE, or as supportive or nonsupportive of *L. monocytogenes* growth, because consumer handling and intended use may not always align (WHO 2022). Accordingly, the purpose of the present review is not to replace existing broad discussions of *L. monocytogenes* control, but to provide a RTE meat‐focused synthesis of thermal and nonthermal intervention studies.

Therefore, this scoping review aimed to map and synthesize the contributions of both thermal and nonthermal interventions to the inactivation of *L. monocytogenes* in RTE meats. The review examined reported inactivation outcomes, mechanistic explanations, and recurring methodological limitations across a range of intervention types, from conventional heat treatments to emerging nonthermal technologies. Rather than treating RTE meat products as a microbiologically uniform category, this review uses the RTE designation as a practical framework for interpreting intervention studies in products intended for direct consumption.

## 2 Methods

### 2.1 Research Questions and Eligibility Criteria

The PICO (population, intervention, comparison, and outcome) framework was used to define the research question and identify relevant records (Higgins et al. 2019). This scoping review was conducted and reported in accordance with the PRISMA Extension for Scoping Reviews (PRISMA‐ScR) (Tricco et al. 2018). Because the available literature spans diverse product types, intervention conditions, and outcome reporting practices, this study was designed as a scoping review. The aim was to map the available evidence and characterize the range of study designs and reported outcomes, rather than to generate a pooled quantitative estimate or formal performance ranking across technologies. The primary question addressed by this review was “What magnitude of log reduction in *L. monocytogenes* contamination is achieved by thermal and nonthermal technologies when applied to RTE meats, compared with untreated controls, and how do processing conditions influence this effect?”

### 2.2 Search Strategy and Data Source

The literature search was conducted in August 2025 using three electronic bibliographic databases: Scopus, PubMed, and Web of Science Core Collection. Search strategies were developed around three main concepts: RTE meats, *L. monocytogenes*, and microbial inactivation, and were detailed in Table 1. Studies published in languages other than English were excluded during screening due to limited translation resources. Grey literature that has not undergone peer review for publication was not included in this study to avoid data validity concerns and data duplication because high‐quality theses and reports were likely to be published in peer‐reviewed journals.

**TABLE 1:** The search algorithm used for each electronic database.

Database	Search syntax	# of Records
Scopus	TITLE‐ABS‐KEY ((listeria) OR (monocytogenes) OR (listeriosis) OR (“L.monocytogenes”)) AND TITLE‐ABS‐KEY (( deli ) OR ( delis) OR ( delicatessen) OR ( charcuterie ) OR ( cold meat* ) OR ( cold cut* ) OR ( lunch meat* ) OR ( luncheon meat* ) OR ( sandwich* ) OR ( salami* ) OR ( sliced salami* ) OR ( pepperoni ) OR ( chorizo ) OR ( bologna ) OR ( baloney ) OR ( boloney ) OR ( polony ) OR ( sliced corned beef ) OR ( sliced ham ) OR ( sliced smoked ham ) OR ( head cheese ) OR ( sliced veal ) OR ( sliced roast beef ) OR ( mortadella ) OR ( smoked meat ) OR ( pastrami ) OR ( beef pastrami ) OR ( turkey pastrami ) OR ( sliced tongue ) OR ( sliced turkey breast ) OR ( sliced chicken breast ) OR ( chicken breast supreme ) OR ( sliced mock chicken ) OR ( pancetta ) OR ( prosciutto ) OR ( ham off the bone ) OR ( chicken roll ) OR ( roast beef )) AND TITLE‐ABS‐KEY (( “reduction” ) OR ( “removal” ) OR ( “inactivation” ))	319
Web of Science	ALL = ((listeria) OR (monocytogenes) OR (listeriosis) OR (“L.monocytogenes”)) AND ALL = (( deli ) OR ( delis) OR ( delicatessen) OR ( charcuterie ) OR ( cold meat* ) OR ( cold cut* ) OR ( lunch meat* ) OR ( luncheon meat* ) OR ( sandwich* ) OR ( salami* ) OR ( sliced salami* ) OR ( pepperoni ) OR ( chorizo ) OR ( bologna ) OR ( baloney ) OR ( boloney ) OR ( polony ) OR ( sliced corned beef ) OR ( sliced ham ) OR ( sliced smoked ham ) OR ( head cheese ) OR ( sliced veal ) OR ( sliced roast beef ) OR ( mortadella ) OR ( smoked meat ) OR ( pastrami ) OR ( beef pastrami ) OR ( turkey pastrami ) OR ( sliced tongue ) OR ( sliced turkey breast ) OR ( sliced chicken breast ) OR ( chicken breast supreme ) OR ( sliced mock chicken ) OR ( pancetta ) OR ( prosciutto ) OR ( ham off the bone ) OR ( chicken roll ) OR ( roast beef )) AND ALL = (( “reduction” ) OR ( “removal” ) OR ( “inactivation” ))	567
PubMed	(((listeria) OR (monocytogenes) OR (listeriosis) OR (“L.monocytogenes”)) AND ((deli) OR (delis) OR (delicatessen) OR (charcuterie) OR (cold meat*) OR (cold cut*) OR (lunch meat*) OR (luncheon meat*) OR (sandwich*) OR (salami*) OR (sliced salami*) OR (pepperoni) OR (chorizo) OR (bologna) OR (baloney) OR (boloney) OR (polony) OR (sliced corned beef) OR (sliced ham) OR (sliced smoked ham) OR (head cheese) OR (sliced veal) OR (sliced roast beef) OR (mortadella) OR (smoked meat) OR (pastrami) OR (beef pastrami) OR (turkey pastrami) OR (sliced tongue) OR (sliced turkey breast) OR (sliced chicken breast) OR (chicken breast supreme) OR (sliced mock chicken) OR (pancetta) OR (prosciutto) OR (ham off the bone) OR (chicken roll) OR (roast beef))) AND ((“reduction”) OR (“removal”) OR (“inactivation”))	225

### 2.3 Relevance Screening

All records identified from the database searches were imported into Rayyan (http://rayyan.ai) for relevance screening. Screening was conducted by two experts: a food engineer specializing in thermal and nonthermal processing technologies for microbial safety, and a food microbiologist specializing in microbial testing and safety control.

Duplicate citations across databases were removed using Rayyan's deduplication function. Relevant articles were selected through a two‐phase process comprising: (i) a preliminary screening based on titles and abstracts, and (ii) an advanced review based on full texts. Studies were included if they evaluated the effect of thermal or nonthermal technologies on RTE meats. Studies were excluded if they: (i) did not involve RTE meats (e.g., raw/partially cooked products), (ii) did not report microbial inactivation data, or (iii) applied thermal or nonthermal technologies in combination with other treatments such as antimicrobial treatments (e.g., essential oils) except for salt and nitrite, which were treated as formulation‐related ingredients commonly present in RTE meat products.

## 3 Results and Discussion

### 3.1 Search Results and Study Characteristics

**FIGURE 1:**
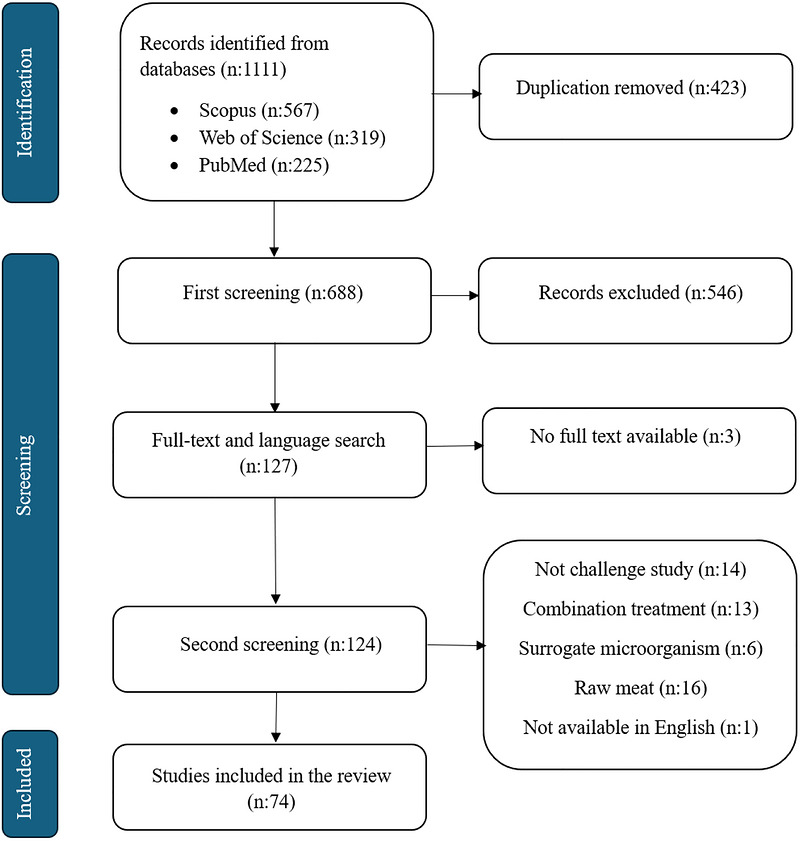
PRISMA flow diagram for scoping review flowchart.

An overview of the scoping review process is presented in Figure 1. The initial search identified 1111 records. After removing duplicates, 688 records were subjected to title and abstract screening, resulting in 124 articles for full‐text review. During the full‐text screening, 50 studies were excluded for the following reasons: use of the wrong population (i.e., not RTE meats), assessment of nonpathogenic microorganisms, publication in a language other than English, or evaluation of thermal and nonthermal technologies in combination with other antimicrobial interventions, such as chemical interventions. Ultimately, 74 studies met the inclusion criteria and were included in the scoping review.

Among the studies included, high‐pressure processing (HPP) was the most frequently investigated technique for inactivating *L. monocytogenes* in RTE meats (29/74, 39.2%), followed by thermal treatments (18/74, 24.2%). Ionizing radiation (gamma, electron beam, and X‐rays), UV light, and pulsed light were evaluated in 9, 8, and 4 studies, respectively. Among the studies included in this review, 31 (31/74, 41.9%) did not report standard deviations for *L. monocytogenes* inactivation results. Additionally, several studies presented inactivation outcomes only as D‐values rather than log reductions. Due to these inconsistencies in data reporting, a meta‐analysis could not be conducted in this study. Notably, 27 studies (27/74, 36.5%) employed only a single *L. monocytogenes* strain in their challenge experiments, limiting the robustness of the findings. Strain background, persistence, and physiological adaptation may contribute to treatment‐specific differences in apparent lethality across studies. The distribution of studies across technologies was uneven, with HPP representing the largest share of the evidence base. Accordingly, differences in the depth of the following sections reflect differences in the volume and maturity of the available literature rather than an a priori ranking of intervention importance.

Key parameters were frequently missed or underreported. Most studies did not provide the fat content (53/74, 71.6%) or water content (51/74, 68.9%) of the RTE meat, despite these factors potentially influencing the efficacy of *L. monocytogenes* inactivation, particularly in treatments such as high‐pressure processing. Nitrite content, salt content, and sample thickness were also often omitted, with 67.1% of studies failing to report nitrite and salt content and 69.9% failing to report product thickness. These omissions may affect the interpretation of both thermal and nonthermal treatment performance.

### 3.2 Thermal Inactivation

Thermal treatments, such as pasteurization and sterilization, remain the most widely used processing technologies in the food industry, primarily to ensure food safety and extend product shelf life. These thermal processes achieve microbial inactivation by applying heat at levels sufficient to destroy pathogenic and spoilage microorganisms, particularly those in their vegetative state (Van Impe et al. 2018).

Heat‐induced injury to vegetative cells is multifaceted, affecting various cellular structures and functions. Damage may occur to the cell wall, cytoplasmic membrane, ribosomes, ribosomal RNA, and enzymes involved in central metabolic pathways, such as the citric acid cycle (Wang et al. 2020). At elevated temperatures, cell death is closely associated with the denaturation of thermally labile proteins, including the α‐ and β‐subunits of RNA polymerase. Additionally, thermal injury is exacerbated by the loss of essential intracellular components, including potassium ions, amino acids, and proteins, resulting from increased membrane permeability (Osek et al. 2022; Van Impe et al. 2018).

#### 3.2.1 The Effect of Thermal Treatment on Microbial Inactivation in RTE Meats

The key characteristics of studies investigating L. monocytogenes inactivation through thermal treatments are reported in Table 2. Notably, 33.3% of the reviewed studies (6 of 18) used only a single *L. monocytogenes* strain in challenge tests, which may limit the generalizability of the results due to strain‐specific thermal resistance.

**TABLE 2:** Summary of published studies on *L. monocytogenes* inactivation using thermal treatment

References	Country	Food type	Processing Conditions	*L. monocytogenes*	Reported outcome
McMinn et al. (2025)	USA	Ham	Temperature: 62.8–71.1°C	LM 101, FSL‐C1‐109, LM 310, V7, and LM 108	up to >7 log
			Time: up to 5 min		
Felício et al. (2011)	Portugal	Alheiras, traditional Portuguese sausage	Temperature: 55–65°C	SL‐F7‐020, FSL‐F7‐088, FSL‐F7‐099, and FSL‐F7‐128	D value: 1.2–8 min
Wang et al. (2016)	Belgium	Sliced ham and sausage	Temperature: 60°C	LFMFP 392, LFMFP 421, and LFMFP 491	4D value: 10.9–25.4 min
Mangalassary et al. (2008)	USA	Turkey bologna	Temperature: 65°C	ATCC 15313	3.5 log CFU/ cm^2^
			Time: 32 s		
Gedela et al. (2007)	USA	Frankfurters	Temperature: 73.9°C	Scott A‐2, V7‐2, 39‐2, and 383‐2	2.9 log
			Time: 1 min		
Gedela et al. (2007)	USA	Deli turkey breast chubs	Temperature: 270°C	Scott A‐2, V7‐2, 39‐2, and 383‐2	∼3.2 log
			Time: 1 min		
Mangalassary et al. (2007)	USA	Turkey bologna	Temperature: 65°C	ATCC 15313	3.5 log CFU/ cm^2^
			Time: 32 s		
Selby et al. (2006)	USA	Bologna	Temperature: 55–65°C	Scott A, F5069, and V7	D value: 1.5–122.3 min
Murphy et al. (2005)	USA	Bologna	Temperature: 100°C (ambient), 131°C (pressurised)	RS#V105, ARS#V67, ARS#V72, ARS#V113, ARS#V125, and LCDC 81–861 4b	Up to 8 log reduction at 100°c at 8 min
					Up to 8 log reduction at 131°c at 2 min
McCormick et al. (2005)	USA	Turkey bologna	Temperature: 65°C	ATCC 15313	4.8 log CFU/g
			Time: 81 s		
Muriana et al. (2004)	USA	Deli turkey	Temperature: 399°C (pre‐package), 93.3°C (post‐package)	Scott A‐2, V7‐2, 39‐2, and 383‐2	Pre‐package: 2.0 to 3.8 log reduction with up to 75 s treatment time
					Post‐package: 1.95 to 3.0 log reduction with up to 3 min treatment time
Gande and Muriana (2003)	USA	Turkey bologna, roast beef, corned beef, and ham	Temperature: 246–399°C for pre‐package	Scott A‐2, V7‐2, 39‐2, and 383‐2	Pre‐package: 1.25 to 3.5‐log
			Time: 1–2 min		Post‐package: 2.0 to 3.75 log
McCormick et al. (2003)	USA	Turkey bologna	Temperature: 61 and 65°C	ATCC 15313	D‐value: 16.2–124 s
Muriana et al. (2002)	USA	Smoked turkey, roast beef, smoked ham	Temperature: 90.6–96.1°C	Scott A‐2, V7‐2, 39‐2, and 383‐2	D‐value: 4.0–416.7 s
			Time: 2–10 min		
Schoeni et al. (1991)	USA	Fermented beaker sausage	Temperature: 48.9–62.8°C	Scott A, V7, LM‐101M, LM‐102M, and LM‐103M	D‐value: 9.13–98.6 min
Mangalassary et al. (2004)	USA	Turkey bologna	Temperature: 60–90°C	ATCC 15313	5D value: 0.35–10 min
			Thickness: 4–20 mm		
Delgado Suárez et al. (2015)	Mexico	Turkey‐based Virginia	Temperature: 75–90°C	ATCC 19114	0.24–3.91 log CFU/cm^2^
			Time: 0–30 s		

Across studies, a clear time‐temperature dependence was observed. McMinn et al. (2025) showed that cooking ham to a core temperature of 71.1°C achieved a ≥6‐log reduction of *L. monocytogenes*. The D‐values of *L. monocytogenes* isolates from alheiras ranged from 7.2 to 9.3 min at 55°C, 1.3 to 2.1 min at 60°C, and 0.6 to 1.8 min at 65°C. These data showed that thermal resistance decreased at higher temperatures (Felício et al. 2011). Similarly, Selby et al. (2006) found D‐values decreased as temperature increased. Also, survival curves at 55, 60, 62.5, and 65°C showed similar D‐values between the two bologna brands, indicating no significant brand‐related differences in thermal resistance at any of the tested temperatures.

Steam‐based pasteurization approaches also showed substantial inactivation. Murphy et al. (2005) compared pasteurization by ambient steam at 100°C or pressurized steam at 131°C. At an initial inoculation level of approximately 10^8^ CFU/cm^2^, *L. monocytogenes* was reduced to below the detection limit on bologna samples following 8 min of ambient steam treatment or 2 min of pressurized steam treatment, with no recoverable survivors reported under the conditions used in that study. While in‐package steam treatments are effective, excess water purge generated during in‐package pasteurization can negatively influence consumer acceptance. Therefore, in addition to in‐package pasteurization, the meat and poultry industry explored alternative interventions that are simple, rapid, cost‐effective, and easily adaptable for high‐volume processing of diverse RTE meats. Prepackaged pasteurization using pressurized steam applies a brief heat treatment before final retail packaging, thereby minimizing water‐purge issues associated with heating meat products in sealed packages. However, because the product may be re‐exposed to the processing environment after treatment, recontamination concerns remain before the final packaging step. Consequently, prepackaging pasteurization must be performed as close as possible to the point of final retail packaging to mitigate this risk (Murphy et al. 2005).

The food matrix also influenced thermal sensitivity. Muriana et al. (2002) compared D‐values for *L. monocytogenes* heated in purge from RTE smoked turkey, roast beef, and smoked ham deli products. The highest D‐values were observed in smoked turkey, whereas the lowest were reported in smoked ham across temperatures ranging from 62.8 to 71°C. These differences may be attributed to surface‐related factors, such as imperfections that can shield cells from heat exposure, as well as the movement of chilled purge toward the product surface, which could enhance microbial protection.

A smaller subset of studies (11.1%, 2 of 18) investigated near‐infrared (NIR) heating as an alternative process to control *L. monocytogenes* in RTE meats. Infrared (IR) radiation, a type of electromagnetic energy, is typically classified into near‐IR (0.76–2 µm), mid‐IR (2–4 µm), and far‐IR (4–1000 µm) regions. IR heating has gained increasing interest as a food processing technology due to its high thermal efficiency, rapid heating rates, and quicker system response compared with conventional heating methods (Anumudu et al. 2024; Krishnamurthy et al. 2008). In ham, Ha and Kang (2014) showed that NIR exposure at 100 µW/cm^2^/nm for 50 s resulted in approximately a 0.67‐log CFU/g reduction in *L. monocytogenes* populations, whereas increasing the intensity to 200 µW/cm^2^/nm for the same duration achieved an additional 2.72‐log CFU/g reduction. In another study, NIR heating at 1.8 kV for 50 s resulted in a 3.38‐log reduction in *L. monocytogenes*, while convective heating at 1.8 kV required 180 s to achieve a comparable level of inactivation (Ha et al. 2012).

Thermal treatments consistently produced strong and relatively predictable reductions of *L. monocytogenes* in RTE meats. Typical D‐values ranged from approximately 7–9 min at 55°C to <2 min at 60°C and <1 min at 65°C, consistent with the expected increase in lethality at higher temperatures. Conventional cooking and steam‐based pasteurization frequently achieved multi‐log reductions under the studied conditions. Matrix‐related factors, including fat content, surface structure, and purge characteristics, influenced apparent thermal resistance, but their effects were generally secondary to time‐temperature intensity. NIR heating produced reductions comparable to convective heating while requiring shorter exposure times in the limited studies available. Overall, the reviewed evidence indicates that thermal treatments remain the most consistent intervention class for reducing *L. monocytogenes* in RTE meats, although cross‐study interpretation is constrained by reporting gaps and the frequent use of single strains in challenge designs.

### 3.3 Nonthermal Technologies

#### 3.3.1 High‐Pressure Processing

High‐pressure processing (HPP), also referred to as high hydrostatic pressure (HHP), is a nonthermal technology widely used to enhance microbial safety and extend the shelf life of food products (Okur 2025). In this technique, pressure levels of up to 1000 MPa are applied to vacuum‐packaged foods placed in a vessel filled with a pressure‐transmitting medium for a defined temperature and treatment time (Balasubramaniam 2021). Since pressure is transmitted instantaneously and uniformly throughout the product, HPP treats foods evenly regardless of size, shape, or composition (Albert et al. 2021). The application of HPP in food manufacturing has increased substantially in recent years (Reesman et al. 2023), with approximately 25% of installed HPP equipment utilized to meat products, particularly ready‐to‐eat and other processed meats (Bolumar et al. 2020; Roobab et al. 2021).

Bacterial membranes are widely recognized as the primary target of pressure‐induced damage. Pressure disrupts membrane integrity, increases permeability, and interferes with membrane‐associated functions (Rux et al. 2020; Yang et al. 2021). Moderate pressures up to 180 MPa can slow microbial growth and may induce sublethal cellular damage. In contrast, pressures exceeding 200 MPa can cause cell death (Bolumar et al. 2021). At these higher pressures (>200 MPa), misfolded proteins or folding intermediates accumulate, which may engage in nonfunctional intermolecular interactions upon decompression, leading to the formation of insoluble protein aggregates (H. Liu et al. 2021; Wang et al. 2022). Significant structural changes have been reported above 400 MPa, resulting from pressure‐mediated disruption of hydrophobic interactions that stabilize protein aggregates (Bolumar et al. 2021).

Food matrix characteristics also affect pressure sensitivity. For example, high fat levels can reduce the efficiency of microbial inactivation during HPP, likely through fat‐derived protective effects around cells and altered pressure‐transmission in the aqueous phase (Koutsoumanis et al. 2022).

##### 3.3.1.1 The Effect of HPP on Microbial Inactivation in RTE Meats

The characteristics of the studies included in the HPP analysis are summarized in Table 3. Reported log reductions among the individual studies ranged from 0 to 7 log. Most studies applied pressures between 300 and 600 MPa, with the highest reaching 800 MPa. Treatment durations were generally below 15 min, and treatment temperatures typically ranged from 4 to 25°C. Eight studies (8/29, 27.6%) included only a single strain when conducting the challenge study. When studies were grouped by comparable pressure levels, a clearer pattern emerged. Treatments below 400 MPa generally resulted in limited inactivation, typically <1 log even with extended holding times, indicating sublethal or marginal effects under those conditions (Chen 2007; Myers et al. 2013; Rubio et al. 2018). In contrast, treatments between 500 and 600 MPa consistently produced higher reductions, most commonly in the range of 1–7 log CFU/g within treatment times of ≤5 min (Table 3). Specifically, in Spanish chorizo sausage, treatment at 349 MPa for 6.25 min resulted in a 0.04‐log reduction, while treatment at 600 MPa for the same treatment time caused a 2.47‐log reduction. Based on predicted isoreduction diagrams results, Hereu et al. (2012) showed that *L. monocytogenes* inactivation, 5 log reduction, was not achievable below 500 MPa for cooked ham and mortadella. Cooked ham required approximately 1 min at 600 MPa or 5 min at 500 MPa to reach a 5‐log reduction. Mortadella required roughly 1 min at 600 MPa or 3 min at 500 MPa. In sliced ham, a 400 MPa treatment for 3 min resulted in a 0.95‐log reduction, whereas 600 MPa for 3 min produced a 4.25‐log reduction (Myers et al. 2013). Porto‐Fett et al. (2010) reported similar results for Genoa salami, comparing the pressure level of 483 and 600 MPa for treatment of up to 5 min.

**TABLE 3:** Summary of the studies included on *L. monocytogenes* inactivation by high‐pressure processing.

References	Country	Food type	Processing conditions	*L. monocytogenes*	Reported outcome
Martillanes et al. (2021)	Spain	Dry‐cured Iberian ham	Pressure: 600 MPa	CECT 933 and 911	3.2 log CFU/mL
			Treatment time: 8 min		
			Temperature: 10°C		
Pérez‐Baltar et al. (2021)	Spain	Dry‐cured ham	Pressure: 450, 600 MPa	S2, S7‐2	0.5–1.3 log CFU/g
			Treatment time: 5,10 min		
			Temperature: 16°C		
Pérez‐Baltar et al. (2019)	Spain	Dry‐cured ham	Pressure: 450 MPa	4 strains (S2 (serotype 1/2a), S4‐2 (serotype 1/2b), S7‐2 (serotype 4b), and S12‐1 (serotype 1/2c))	0.45 log CFU/g
			Treatment time: 10 min		
			Temperature: 18°C		
de Alba et al. (2015)	Spain	Dry‐cured ham	Pressure: 450 MPa	H66a	0.44 log CFU/g
			Treatment time: 10 min		
Montiel et al. (2015)	Spain	Cooked ham	Pressure: 450 MPa	INIA 2530	0.78 log CFU/g
			Treatment time: 5 min		
			Temperature: 6°C		
Serra‐Castelló et al. (2025)	Spain	Cooked ham	Pressure: 400 MPa	CTC1034	∼0.2–3.6 log
			Treatment time: 1–15 min		
Myers, Cannon et al. (2013)	USA	Cured ham	Pressure: 450, 600 MPa	CC 7644, NCTC 10890, ATCC 19112, ATCC 19114, and ATCC 19115	0.70–4.25 log CFU/g
			Treatment time: 3 min		
Porto‐Fett et al. (2010)	USA	Genoa salami	Pressure: 600 MPa	MFS2, MFS102, MFS104, MFS105, MFS110	1.62–5.02 log CFU/g
			Treatment time: 1–5 min		
Hayman et al. (2004)	Australia	Low‐fat pastrami, Strassburg beef, export sausage, and Cajun beef	Pressure: 600 MPa	2542, 2655, 2657, 2340, 2341, 2342, 2343, 2345	∼4 log CFU/g
			Treatment time: 3 min		
Koseki et al. (2007)	Japan	Cooked ham	Pressure: 400–600 MPa	ATCC 19111, ATCC 19118, ATCC 13932, ATCC 15313, and ATCC 35152	Up to 3 log CFU/g
			Treatment time: 1–60 min		
			Temperature: 25°C		
Chen (2007)	USA	Turkey breast meat	Pressure: 300–500 MPa	PSU1, PSU2, PSU9, PSU21, and ATCC 19115	0–7.5 log CFU/g
			Treatment time: 1–2 min		
			Temperature: 1–55°C		
Teixeira et al. (2016)	Canada	Cooked ham	Pressure: 500,600 MPa	FSL J1‐177, FSL C1‐056, FSL N3‐013, FSL R2‐499, and FSL N1‐227	0–4.2 log
			Treatment time: 0–32 min		
			Temperature: −5–32°C		
Rubio et al. (2009)	Spain	Cecina and Salchichón	Pressure: 500 MPa	CECT 932	1.49 log CFU/g
			Treatment time: 5 min		
			Temperature: 18°C		
Pavli et al. (2019)	Greece	Ham	Pressure: 500 MPa	FMCC‐B‐129, FMCC‐B‐131 and FMCC‐B‐133	∼1.2 log CFU/g
			Treatment time: 2 min		
			Temperature: 20°C		
Serra‐Castelló et al. (2021)	Spain	Cooked ham	Pressure: 400 MPa	CTC1011, CTC1034, Scott A	Up to 6.5 log CFU/g
			Treatment time: 0–10 min		
Cava et al. (2020)	Spain	Traditional dry‐cured sausage	Pressure: 600 MPa	1/2a, 1/2b, 1/2c and 4b	∼2 log CFU/g
			Treatment time: 8 min		
			Temperature: 16°C		
Rubio et al. (2018)	Spain	Spanish chorizo sausage	Pressure: 349–600 MPa	CECT935 and 3 strains	Up to 3.71 log CFU/g
			Treatment time: 0–12.53 min		
Hereu et al. (2014)	Spain	Cooked ham and mortadella	Pressure: 400 MPa	CTC1034	Up to 4 log CFU/g
			Treatment time: 5 min		
			Temperature: 15°C		
Stollewerk et al. (2014)	Spain	Dry‐cured ham	Pressure: 600 MPa	CTC1011, CTC1034 and CECT4031	Up to 2 log CFU/g
			Treatment time: 5 min		
			Temperature: 13°C		
Myers, Montoya et al. (2013)	USA	Sliced ham and turkey breast	Pressure: 600 MPa	ATCC 7644, NCTC 10890, ATCC 19112, ATCC 19114, and ATCC 19115	Up to 4.35 log CFU/g
			Treatment time: 3 min		
Hereu et al. (2012)	Spain	Cooked ham and mortadella	Pressure: 300–800 MPa	CTC1034	Up to 7 log CFU/g
			Treatment time: 0–3 min		
			Temperature: 15°C		
Stollewerk et al. (2012)	Spain	Smoked dry‐cured ham	Pressure: 600 MPa	CTC1011, CTC1034 and CECT4031	Up to 1.8 log CFU/g
			Treatment time: 5 min		
			Temperature: 13°C		
Marcos et al. (2008)	Spain	Cooked ham	Pressure: 400 MPa	CTC1010, CTC1011, and CTC1034	∼3 log CFU/g
			Treatment time: 10 min		
			Temperature: 17°C		
Jofré et al. (2008)	Spain	Cooked ham	Pressure: 600 MPa	CTC1010, CTC1011, and CTC1034	∼4 log CFU/g
			Treatment time: 5 min		
			Temperature: 10°C		
Balamurugan et al. (2018)	Canada	Cooked sausages	Pressure: 600 MPa	08‐5578, Li0512, Li0529, and ATCC 19115	∼7 log CFU/g
			Treatment time: 3 min		
			Temperature: 4°C		
Lavieri et al. (2014)	USA	Restructured ham	Pressure: 400, 600 MPa	Scott A NADC 2045 serotype 4b, H7969 serotype 4b, H7962 serotype 4b, H7596 serotype 4b, and H7762 serotype 4b	Up to 3 log CFU/g
			Treatment time: 1, 4 min		
			Temperature: 12°C		
Bover‐Cid et al. (2015)	Spain	Dry‐cured ham	Pressure: 347–852 MPa	CTC1034	0.92–7.96 log
			Fat: 10–50.36%		
Fonberg‐Broczek et al. (2004)	Spain	Ham	Pressure:100–500 MPa	NCTC 11994	D‐value: 2.40–31.80 min
Bover‐Cid et al. (2019)	Spain	Cooked ham	Pressure: 600 MPa	CTC1034, 12MOB045LM, 12MOB089LM	∼2.5 log CFU/g
			Treatment time: 3 min		

Strain‐dependent variability was observed. Serra‐Castelló et al. (2021) reported that *L. monocytogenes* resistance to high‐pressure processing varied among serotypes. At 400 MPa for treatment times ranging from 0 to 10 min, inactivation levels reached up to 4 log CFU/g for strains CTC1034 and Scott A, and up to 7 log CFU/g for strain CTC1011. When dry‐cured ham was treated at 450 MPa for 10 min, *L. monocytogenes* strains S2 and S7‐2 were reduced by 0.8 log CFU/g for dry‐cured ham. These reductions increased to 1.3 and 1.5 log CFU/g when treated at 600 MPa for 5 min (Pérez‐Baltar et al. 2021).

In the reviewed HPP studies, salt and nitrite concentrations did not consistently alter the immediate inactivation of *L. monocytogenes* under the pressure conditions tested. For ham and turkey, Myers et al. (2013) examined different salt concentrations (1.8% and 2.4%) and nitrite concentrations (0 and 500 ppm) at 600 MPa for 3 min and observed reductions of approximately 3.85–4.35 log CFU/g without significant differences attributable to salt or nitrite concentration, whereas 400 MPa for 3 min provided less than 1 log CFU/g reduction, indicating that pressure level remained the dominant driver of immediate inactivation under those conditions. Similar results were reported for sliced ham treated at 400 or 600 MPa for 3 min across nitrite concentrations of 0, 100, and 200 ppm (Myers et al. 2013). Lavieri et al. (2014) investigated RTE ham containing 0, 50, or 100 ppm nitrite and found that treatment at 600 MPa for 4 min reduced *L. monocytogenes* to below the detection limit immediately after treatment, independent of nitrite concentration. However, ham formulations lacking nitrite showed faster subsequent growth during storage than formulations containing nitrite, irrespective of HPP treatment. These findings suggest that nitrite may contribute more to suppression of recovery or outgrowth during storage than to the immediate reduction achieved by HPP under the conditions studied. This distinction is important because sodium nitrite remains a multifunctional curing ingredient in meat systems, but current replacement or reduction strategies increasingly include plant‐based nitrate/nitrite sources and other formulation‐based approaches such as organic acids or related hurdle combinations rather than a single universal substitute.

There were few studies focused on the initial sample temperature of *L. monocytogenes* inactivation. Inoculated turkey breast meat samples were vacuum‐packaged and subjected to pressure treatments at 300 MPa for 2 min, and at 400 and 500 MPa for 1 min each, with initial sample temperatures of 1, 10, 20, 30, 40, 50, and 55°C. *L. monocytogenes* exhibited the highest‐pressure resistance at temperatures between 10°C and 30°C. As the temperature decreased below 10°C or increased above 30°C, pressure sensitivity increased (Chen 2007).

Product composition also influenced HPP performance. Hereu et al. (2012) compared the effect of HPP on *L. monocytogenes* inactivation in the cooked ham (4.55% fat) and mortadella (17.08% fat). It was found that differences in HPP inactivation of *L. monocytogenes* were observed at pressures ranging from 373 to 550 MPa, with slower inactivation rates in mortadella compared with cooked ham. The higher lipid content in mortadella (17.08%) may provide some protection to *L. monocytogenes* against the lethal effects of high pressure. In dry‐cured ham, Bover‐Cid et al. (2015) investigated the effect of different fat content (10%–50%) on *L. monocytogenes*. These results indicate that fat content influenced the lethality of high‐pressure processing, and its effect varies with pressure intensity. At pressures above 650 MPa, high fat levels can provide piezoprotection, reducing microbial inactivation. Conversely, at lower pressure levels, products with higher fat content exhibited greater *L. monocytogenes* reductions, likely due to structural changes within the matrix.

Across the reviewed studies, HPP showed variable but often substantial inactivation of *L. monocytogenes* in RTE meats. Most treatments used 300–600 MPa for less than 15 min at 4–25°C, and reported reductions ranged from minimal change to approximately 7 log depending on treatment intensity and product context. Inactivation generally increased sharply above 500 MPa, with several studies reporting multi‐log reductions at 600 MPa within a few minutes. Strain variability contributed to different pressure sensitivities, and matrix factors such as fat content and product type influenced lethality. Higher fat levels sometimes provided piezoprotection at ≥650 MPa, while effects were mixed at lower pressures. Salt and nitrite levels did not significantly affect outcomes, and products were most pressure‐resistant at 10–30°C. The available evidence therefore supports HPP as a mature and potentially effective nonthermal intervention, but its performance is not uniform across products or treatment settings and should be interpreted in relation to pressure level, strain background, and product composition. On the industry side, the HPP equipment market has substantial growth, reaching a value of USD 885.5 million in 2024. This upward trend is expected to continue, with the market forecasted to attain USD 1863.1 million by 2030, reflecting a compound annual growth rate (CAGR) of 13.2% over the 2025–2030 period (Roy 2025). Nevertheless, several factors continue to limit the broader implementation of HPP technology in the meat industry. One of the primary constraints is the substantial equipment cost associated with HPP systems, which can be prohibitive for small and medium‐sized enterprises (Khojasteh et al. 2025). In addition to economic and technical barriers, HPP can alter important sensory characteristics of food products, including color, flavor, and texture (Sun et al. 2025). Therefore, optimization of processing conditions is necessary to reduce undesirable quality changes, enhance consumer acceptance, and support the wider adoption of HPP in the meat industry.

#### 3.3.2 Pulsed Light

Pulsed light (PL) processing, also known as pulsed UV light (PUV), intense pulsed light (IPL), or high‐intensity pulsed light (HIPL), is one of the newest nonthermal technologies evaluated for food preservation. The FDA approved the application of PL in foods in 1996, with a maximum accumulated UV dose (fluence) of 12 J/cm^−2^, emission spectra ranging from 200 to 1100 nm, and pulse durations of ≤2 ms (Salazar‐Zúñiga et al. 2023). Inert gas flash lamps, predominantly xenon‐based, are used to generate extremely short (microsecond‐scale) high‐power pulses of broad‐spectrum light.

Microbial inactivation by PL is a nonselective, multitarget process, with photochemical, photothermal, and photophysical effects playing a role. Photochemical inactivation is the primary mechanism and is driven by UV‐C absorption by microbial DNA, leading to the formation of pyrimidine dimers that inhibit DNA replication (photochemical effect) (Pollock et al. 2017). At higher power levels, the infrared (IR) component contributes to damage through localized heating and cell rupture (Albert et al. 2021; Mandal et al. 2020). Photophysical effects include alterations in cell morphology, membrane disruption, cytoplasmic damage, and leakage of intracellular components (Jaiswal et al. 2024).

##### 3.3.2.1 The Impact of Pulsed Light on Microbial Inactivation in Ready‐To‐Eat (RTE) Meats

The characteristics of studies on *L. monocytogenes* inactivation by PL treatment were summarized in Table 4. Reported log reductions among the individual studies ranged from 0.4 to 2.3 log. Two out of the four studies (50%) used only a single strain when conducting the challenge study. Fluence levels ranged from 1.74 to 16.11 J/cm^2^. Except for Rajkovic et al. (2017), all studies produced reductions below 1 log, indicating limited lethality under the studied conditions.

**TABLE 4:** Overview of studies evaluating *L. monocytogenes* inactivation by pulsed light (PL).

References	Country	Food type	Processing Conditions	*L. monocytogenes*	Reported outcome
Fracari et al. (2024)	Brazil	Mortadella	Voltage: 3 kV	CCT 7474	0.4–1.37 log CFU/cm^2^
			Distance: 10.95 cm		
			Fluence: 2.64–6.57 J/cm^2^		
			Pulse width: 1260, 2520 µs		
			Number of pulse:1–3		
Borges et al. (2023)	Portugal	Sliced salpicão	Voltage: 1828–3000 V	3 strain coctails	0.72–1.43 log CFU/g
			Distance: 2.6–5.4 cm		
			Fluence: 1.74–16.11 J/cm^2^		
Rajkovic et al. (2017)	Belgium	Fermented salami	Fluence: 3, 15 J/cm^2^	LFMFP 034, 235, 392.447	1.16–2.28 log CFU/g
			Voltage: 3 kV		
			Number of pulse:1,5		
Hierro et al. (2011)	Spain	Cooked ham and bologna slices	Fluence: 0.7–8.4 J/cm^2^	CIP 103575	0.37–1.15 log CFU/cm^2^

*Note*: Fluence, voltage, distance, pulse width, and pulse number differed across studies; values were shown as reported and were not normalized across experimental systems.

Rajkovic et al. (2017) investigated the effect of pulsed light (PL) fluence (3 and 15 J/cm^2^) by applying different numbers of pulses (1 and 5 pulses) on sliced fermented salami. PL treatments were applied either 1 or 30 min after inoculation. Higher fluence resulted in higher *Listeria* reduction (2.24‐log at 3 J/cm^2^ vs. 2.28‐log at 15 J/cm^2^), although this difference was not statistically significant (*p* > 0.05). The timing of PL treatment relative to contamination was more influential. When PL was applied for 1 min post‐inoculation, only 1.10–1.16 log reductions were achieved, whereas applying PL 30 min after inoculation resulted in 2.24–2.28 log reductions. These findings suggested that additional pulses did not meaningfully increase inactivation and that microbial attachment time influences PL effectiveness.

PL inactivation curves often exhibit a sigmoidal curve, similar to continuous UV light (Jaiswal et al. 2024; Rajkovic et al. 2017). A plateau in microbial reduction has been reported once a critical energy threshold is reached, indicating diminished incremental inactivation at higher fluences (Rajkovic et al. 2017). Fracari et al. (2024) reported similar trends on sliced mortadella treated at fluences of 2.64–6.57 J/cm^2^ by varying pulse width (1260–2520 µs) and number of pulses (1–3). Reductions ranged from 0.43 to 1.44 log. The 4.38 J/cm^2^ treatment produced a significantly greater reduction (*p* < 0.05) than the 2.64 J/cm^2^ treatment, likely due to the higher number of pulses. Among the lower‐fluence treatments, the 4.38 J/cm^2^ treatment resulted in a significantly greater reduction (*p* < 0.05) compared with the 2.64 J/cm^2^ treatment. This difference was likely associated with the higher number of pulses delivered. In another study, vacuum‐packaged ham and bologna slices were surface‐inoculated with *L. monocytogenes* and subjected to PL treatments at 0.7, 2.1, 4.2, and 8.4 J/cm^2^ (Hierro et al. 2011). The highest fluence (8.4 J/cm^2^) resulted in reductions of 1.78 log CFU/cm^2^ on cooked ham and 1.11 log CFU/cm^2^ on bologna. A response surface experimental design was applied to evaluate the effects of voltage (1828–3000 V) and distance from the light source (2.6–5.0 cm), yielding different fluences (1.74–16.11 J/cm^2^) on traditional cured smoked meat sausage (Borges et al. 2023). A reduction of 1.58 log CFU/g in *L. monocytogenes* was achieved at a fluence of 5.31 J/cm^2^. Also, for fluences below 5 J/cm^2^ *, L. monocytogenes* reductions increased in a dose‐dependent manner, displaying an approximately linear relationship. However, at fluences above 5 J/cm^2^, a tailing effect was observed, indicating diminished incremental inactivation at higher doses that also supports the result of Rajkovic et al. (2017).

According to the studies reviewed, pulsed light produced modest inactivation of *L. monocytogenes* in RTE meats, with reductions ranging from 0.4 to 2.3 log. Fluences ranged from 1.74 to 16.11 J/cm^2^, and half of the studies used only a single *L. monocytogenes* strain. Most treatments produced modest reductions, often below 1 log, indicating limited lethality under the studied conditions. Only one study achieved ≥2‐log reductions, and this occurred when PL was applied 30 min after contamination, suggesting that microbial attachment time affects treatment performance. Inactivation increased with fluence at lower doses but plateaued at higher intensities, showing a sigmoidal dose‐response pattern. Matrix effects were evident, with different RTE products showing variable sensitivity. Overall, PL produced modest and condition‐dependent reductions of *L. monocytogenes* in RTE meats, with performance constrained by fluence, attachment conditions, and product surface characteristics.

#### 3.3.3 Ultraviolet Light

Ultraviolet (UV) light has long been used to disinfect surfaces, water, and air, and has recently gained attention in the food industry as a rapid and cost‐effective method for the hygienization of foods (Chiozzi et al. 2022). The U.S. FDA approved UV in 2000 as an inactivation method for controlling microorganisms in foods, water, and beverages. The primary mechanism of microbial inactivation is DNA damage. UV exposure induces the formation of pyrimidine dimers in DNA and RNA, which interfere with DNA replication and ultimately lead to cell death (Iammarino et al. 2022; Kim and Song 2023). Low and medium‐pressure mercury lamps are commonly used to emit UVC wavelengths (200–280 nm). However, these lamps may contain toxic mercury, raising environmental and human health concerns (Iammarino et al. 2022).

##### 3.3.3.1 The Impact of Ultraviolet Light on Microbial Inactivation in RTE Meats

The key characteristics of studies evaluating *L. monocytogenes* inactivation by UV are summarized in Table 5. The applied dose varied between 5.4 and 800 J/cm^2^ across studies. Notably, half of the studies (3/6) employed only a single *L. monocytogenes* strain in their challenge experiments, limiting the strength of the conclusions. Across the reviewed studies, UV treatment resulted in limited but measurable inactivation of *L. monocytogenes* on RTE meat surfaces, with reductions generally ranging from approximately 0.54 to 1.97 log CFU/g. When studies were grouped by comparable dose levels, a clearer trend emerged. At lower doses (<50 mJ/cm^2^ or equivalent), inactivation was typically <1 log, indicating insufficient energy delivery for substantial microbial reduction. At moderate doses (approximately 50–300 mJ/cm^2^), reductions more commonly ranged between 1 and 2 log CFU/g, although variability across studies remained pronounced.

**TABLE 5:** Summary of research on *L. monocytogenes* inactivation through ultraviolet (UV) light exposure

References	Country	Food type	Processing Conditions	*L. monocytogenes*	Reported outcome
Kim and Kang (2020)	South Korea	Sliced deli meat	Dose: 5.4–21.6 mJ/cm^2^	3‐strain cocktail (ATCC 15313, ATCC 19111, and ATCC 19115	0.54–1.12 log CFU/cm^2^
			UVC range: 240–280 nm		
			Distance:3 cm		
			Time <21.6 s		
Kim et al. (2025)	South Korea	Deli turkey breast	Dose: 112.3–786.3 mJ/cm^2^	ATCC 19112	0.6–1.8 log CFU/g
Lee et al. (2025b)	South Korea	Prosciutto	UVC range: 265–275 nm	KCCM 40307	1.1–2.0 log CFU/g
			Time: 10–50 s		
Ha and Kang (2015)	South Korea	Sliced ham	Intensity: 1.85 mW/cm^2^	ATCC 15313, ATCC 19111, and ATCC 19115	1.0–1.55 log
			Time: 10–70 s		
Chun et al. (2009)	South Korea	Sliced ham	Dose: 1000–8000 J/m^2^	ATCC 19111	1.22–2.74 log CFU/g
C. H. Sommers et al. (2009)	USA	Frankfurters	Dose: 1–4 J/cm^2^	F4561, H7762, and H7764	1.31–1.97 log

*Note*: Dose values are reported in the units used by the original studies; differences in wavelength, dose unit, and exposure geometry limit direct quantitative comparison across studies.

Kim and Kang (2020) tested UV light at 280 nm at different doses (5–21.6 mJ/cm^2^) on *L. monocytogenes* inactivation in deli meat, reporting 0.54–1.12 log CFU/cm^2^. The relationship between UV dose and inactivation was linear. Chun et al. (2009) treated sliced ham with UVC at doses of 1000–8000 J/m^2^, achieving 1.22–2.74‐log reductions. Kim et al. (2025) used 222 nm UV light by applying different dose levels (112.3–786.3 mJ/cm^2^) on *L. monocytogenes* inactivation in RTE turkey breast. The log reduction level ranged from 0.6 to 1.8 log CFU/g. In this study, lower inactivation compared with liquid buffer or food‐contact surfaces was attributed to food‐matrix effects, including chemical composition and surface topography (Chun et al. 2009). Consequently, variations in food composition and surface structure can critically influence the performance of light‐based technologies in reducing microbial contamination (Allende et al. 2006).

Lee et al. (2025) used different UV wavelengths (265 and 275 nm) and different intensities (10 and 50 W) on prosciutto. Neither wavelength nor intensity significantly affected inactivation for treatment times up to 50 s. Maximum reductions reached approximately 2 log. Sommers et al. (2009) reported 1.31, 1.49, and 1.93 log reductions on frankfurters at fluences of 1, 2, and 4 J/cm^2^, respectively. Ha and Kang (2014) observed a 1.55‐log reduction on ham slices after 70 s of UVC exposure (0.13 J/cm^2^). Overall, UV light provided measurable but generally modest reductions of *L. monocytogenes* on RTE meats, and its performance was strongly influenced by dose, wavelength, product topography, and shielding by the food matrix.

The practical application of UV‐C light in the food industry is limited by its low penetration depth. Moreover, antimicrobial doses are often associated with oxidative degradation, which can negatively affect meat quality (Monteiro et al. 2023). These drawbacks represent key constraints for its widespread industrial adoption. Nonetheless, given its low cost and ease of implementation, UV‐C treatment can be effectively integrated with other preservation strategies—such as high‐pressure processing, cold plasma, near‐infrared heating, and modified atmosphere packaging—to enhance overall efficacy and facilitate its use as part of a multihurdle approach in industrial food preservation (Monteiro et al. 2023; Tchonkouang et al. 2023).

#### 3.3.4 Ionizing Radiations (Gamma Irradiation, Electron Beam, and X‐Ray)

Ionizing radiation, including gamma rays, electron beams, and X‐rays, is an effective nonthermal pasteurization technology. Exposure of food to ionizing radiation, either in the form of electromagnetic energy (γ‐rays) or charged particles (electron beams), can enhance microbial safety and extend shelf life (Jayathilakan et al. 2017). Radioisotopes such as caesium‐137 and cobalt‐60 are commonly used as sources of γ‐radiation, whereas electron beams are generated using a linear accelerator (Albert et al. 2021). Microbial inactivation by irradiation occurs through both direct and indirect mechanisms. Direct effects include photon‐induced single and double‐strand breaks in DNA, while indirect effects result from DNA damage caused by radiolysis products such as hydroxyl radicals (H. J. Kim and Song 2023; Li et al. 2025). Although the antimicrobial efficacy of γ‐rays and electron beams is comparable, electron beams enable significantly higher dose rates (10^3^–10^5^ Gy/s) compared with γ‐rays (0.01–1 Gy/s), thereby reducing treatment time. In contrast, γ‐rays offer greater penetration depth within the food matrix (60–80 cm) than electron beams (8–10 cm) (“Food Irradiation Using X‐Rays,” 2005). These performance differences influence the selection of irradiation type depending on product thickness, density, and packaging configuration.

##### 3.3.4.1 The Impact of Ionizing Radiation (Gamma Irradiation, Electron Beam, and X‐Ray) on Microbial Inactivation in RTE Meats

The key characteristics of studies evaluating gamma irradiation, electron beam, and X‐ray treatments against *L. monocytogenes* in RTE meats are summarized in Table 6. Notably, two studies (2/9, 22.2%) used only a single *L. monocytogenes* strain in their challenge experiments. All included studies reported reductions greater than 1 log, indicating consistent lethality across products and irradiation formats under the evaluated conditions.

**TABLE 6:** Overview of studies assessing *L. monocytogenes* inactivation by ionizing radiations (gamma irradiation, electron beam, and X‐ray)

References	Country	Food type	Irradiation Source	Processing Conditions	*L. monocytogenes*	Reported outcome
Cho and Ha (2019)	South Korea	Ham	X‐ray	Dose: 0.2–0.8 kGy	3 strains (ATCC 15313, ATCC 19111, and ATCC 19115)	2.8–6.9 log CFU/g
Silva et al. (2021)	Brazil	Cooked ham	Gamma radiation	Dose: upto 2.0 kGy	ATCC 19117	Up to 7 log
				Nitrite (0–150 mg/kg)		
Sommers and Fan (2003)	USA	Fine‐Emulsion Sausage	Gamma radiation	Dose: upto 3.0 kGy	H7595, H7762, H7969, and H7962	Up to 7 log
Jin et al. (2009)	USA	Sliced turkey deli	Gamma radiation	Dose: 1–2 kGy	H7762, H7764, F4249, and F4561	1.58–3.01 log CFU/cm^2^
Zhu et al. (2008)	USA	Turkey hams and breast rolls	E‐beam	Dose: 1.0–2.5 kGy	Scott A, H7969, H7596, H7762, and H7962	1.5–4.7 log in breast rolls
						2.0–5.5 log in ham
Foong et al. (2004)	USA	Frankfurters, ham, roast beef, bologna, smoked turkey	E‐beam	Dose: 0–4	1/2a H7764, 4b H7969, 4b H7962, and OB90393	Up to >8 log
Clardy et al. (2002)	USA	Ham and cheese	Gamma radiation	Dose: 1.15–5.4 kGy	16397, 0733, and 1992	Up to 6.1 log
Cambero et al. (2012)	Spain	Iberian dry‐cured ham, dry‐smoked beef	E‐beam	Dose: 1.5 kGy	Scott A	Up to 3.6 log
Song et al. (2011)	South Korea	Ham	E‐beam	Dose: 1.6 kGy	ATCC15313, ATCC19115	2.4 log CFU/g

Silva et al. (2021) reported that 2.0 kGy of Co‐60 gamma irradiation reduced *L. monocytogenes* ATCC 19117 to below the detection limit in cooked ham immediately after treatment, although regrowth occurred during refrigerated storage in the absence of nitrite. Up to 7 log reduction was observed at 2 kGy. Clardy et al. (2002) demonstrated that gamma irradiation of frozen ham and cheese sandwiches reduced *L. monocytogenes* by ∼5 logs at 3.9 kGy, while 5.9 kGy reduced populations to below the detection limit throughout 39 days of storage under the conditions of that study. Sommers and Fan (2003) showed that Cs‐137 irradiation of frankfurters and bologna inoculated with a four‐strain cocktail resulted in nearly 2.5‐log reductions at 1.5 kGy, with ≥5‐log reductions and growth suppression achieved at 3.0 kGy; D‐values ranged from 0.53 to 0.59 kGy.

Electron beam studies showed similar patterns. Song et al. (2011) applied a 1 MeV electron beam treatment to RTE ham, finding a 2.4‐log reduction at 2.0 kGy against a two‐strain cocktail of *L. monocytogenes*. Similarly, Jin et al. (2009) reported reductions of approximately 1.5 logs at 1.0 kGy and 3.0 logs at 2.0 kGy in deli turkey inoculated with a four‐strain cocktail. Zhu et al. (2008) observed sample‐based outcomes, with 2.5 kGy of electron beam reducing *L. monocytogenes* by 4.7 logs in turkey breast rolls and 5.5 logs in turkey ham; reported D‐values were 0.47–0.52 kGy, respectively. Foong et al. (2004) found that 1.5–2.0 kGy produced 3‐log reductions across six RTE meat types, while 2.5–3.0 kGy achieved 5‐log reductions; the average D‐value was 0.44 kGy. Cambero et al. (2012) reported 3.1–3.6 log reductions at 1.5 kGy in Iberian. Finally, Cho and Ha (2019) investigated X‐ray irradiation of sliced ham, showing that 0.2 kGy achieved a 2.8‐log reduction, while 0.6–0.8 kGy reduced populations below the detection limit.

In general, ionizing radiation consistently produced strong reductions of *L. monocytogenes* in RTE meats, with all studies achieving more than a 1‐log reduction and many reaching 3–5 logs depending on dose and product type. Most effective doses ranged from 1.5 to 3.0 kGy, which typically resulted in ≥3‐log reductions, while doses of ≥3.5 kGy reliably achieved ≥5‐log reductions or reductions to below the detection limit under the reported study conditions. Reported D‐values were generally below 0.6 kGy, with sliced and simpler matrices showing values closer to 0.4–0.5 kGy. Different sources showed similar antimicrobial efficacy, although penetration depth and dose rate varied by technology. More heterogeneous products, such as frankfurters and emulsified meats, required slightly higher doses for equivalent lethality, whereas sliced and uniformly structured meats responded more consistently. The reviewed evidence indicates that ionizing radiation was among the most reliable nonthermal interventions in terms of achieving substantial reductions across different RTE meat products. However, immediate count reduction should be distinguished from longer‐term suppression of recovery or regrowth during storage.

Gamma and X‐ray irradiation are characterized by their high penetration capacity, making them particularly suitable for treating bulky or densely packed food products. In contrast, electron beam (e‐beam) irradiation has a relatively shallow penetration depth and is therefore more appropriate for surface decontamination applications. Compared with conventional food processing methods, irradiation is considered an environmentally friendly and cost‐effective technology. Its advantages include minimal impact on sensory attributes and nutritional quality, broad applicability across different food matrices, high processing efficiency, reduced risk of secondary contamination, and the ability to degrade or eliminate certain hazardous compounds (Khojasteh et al. 2025). Ionizing irradiation technologies, including gamma rays, X‐rays, and electron beam (e‐beam), offer strong potential for industrial application in ready‐to‐eat (RTE) foods.

#### 3.3.5 Cold Plasma

Cold plasma is a relatively new nonthermal sterilization technology compared with the other processing methods described in this review. Plasma is considered the fourth state of matter and is generated by supplying energy to a gas within an electromagnetic field. It comprises a mixture of reactive species, UV photons, ions, electrons, and neutral atoms and molecules (Kim and Song 2023; Oliulla et al. 2024).

Based on the thermodynamic equilibrium between electrons and ions, plasmas are classified as either thermal (hot) or nonthermal (cold). In contrast to thermal plasma, cold plasma is characterized by a much higher electron temperature (T_e_) compared with both the bulk gas temperature (T_g_) and the temperature of the constituent ions. For this reason, cold plasma is also referred to as nonequilibrium plasma (Misra and Jo 2017). Atmospheric pressure systems, which are suitable for food processing, require much higher voltages, typically in the kilovolt range (Paulsen et al. 2022).

The microbial inactivation mechanism of cold plasma technology occurs primarily through the generation of reactive oxygen species (ROS). Also, microbial inactivation occurs due to reactive nitrogen species, UV radiation, energetic ions, and charged particles (Ghazali et al. 2025; Koker et al. 2020). The reactive components may damage key cellular components of pathogenic microorganisms, including nucleic acids, proteins, and cell envelopes (Coutinho et al. 2018). Recent mechanistic studies indicate that, for Gram‐positive bacteria including *L. monocytogenes*, oxidative injury and leakage of intracellular components are major features of cold plasma inactivation, whereas the extent of visible cell‐wall damage may be lower than that observed in Gram‐negative bacteria (Olatunde et al. 2019). In *L. monocytogenes*, plasma exposure has been associated with increased membrane permeability, leakage of nucleic acids and proteins, increased conductivity of the surrounding medium, reduced activity of membrane‐associated enzymes such as Na+/K+‐ATPase and malate dehydrogenase, and alterations in enzyme secondary structure (Qian et al. 2022). A recent multi‐omics study further suggested that sublethal cold plasma exposure in *L. monocytogenes* is accompanied by intracellular oxidative stress, perturbation of cytoplasmic pH homeostasis, altered transmembrane transport, reconfiguration of central carbon metabolism, and activation of repair‐associated responses, although these observations were made under specific experimental conditions and should be interpreted accordingly (Pan et al. 2023).

##### 3.3.5.1 The Effect of Cold Plasma on Microbial Inactivation in RTE Meats

Studies evaluating *L. monocytogenes* inactivation by cold plasma have reported reductions up to 4 log (Table 7). Six of these studies (6 of 8) relied on a single *L. monocytogenes* strain for challenge testing. When grouped by treatment duration and process intensity, short exposures (<2–3 min) generally resulted in <1 log reduction, whereas intermediate treatments (approximately 3–10 min) typically achieved 1–3 log reductions.

**TABLE 7:** Characteristics of studies investigating *L. monocytogenes* inactivation by cold plasma (CP) treatment

References	Country	Food type	Processing Conditions	*L. monocytogenes*	Reported outcome
Calvo et al. (2020)	Spain	Chorizo	Gas flow: 10 L/min	ATCC 15313	Chorizo: 0.37–1.04 log
		Salami	Voltage: 2 kV		Salami: 0.32–0.80 log
		Bacon	Temperature <40°C		Bacon: 0.38–1.00 log
			Treatment time: 4–15 min		
Gök et al. (2019)	Turkiye	Pastirma	Gas flow: 1 L/min	ATCC 51774	0.32–0.83 log CFU/cm^2^
			Treatment time: 3–5 min		
			Gas type: Argo, oxygen		
			Voltage: 25 kV		
Lis et al. (2018)	Brazil	Ham	Voltage: 6.4–10 kV	Field strain	Up to 1.2 log CFU/g
			Frequency: 2–10 kHz		
			Air humidity:45%–90%		
			Treatment time: 10–20 min		
Lee et al. (2011)	China	Cooked chicken breast and ham	Treatment time: 2 min	KCTC 3596	Up to 4 log CFU/g
			Gas flow: 7 L/min		
Song et al. (2009)	South Korea	Ham	Gas flow: 10 L/min	ATCC 19114, 19115, and 19111	Up to 2 log CFU/g
			Treatment time: 0–2 min		
Csadek et al. (2023)	Austria	Cured ham and sausage	Treatment time:2–5 min	NCTC 11994 and 17001	Up to 1.2 log CFU/g
Lee et al. (2025a,b)	South Korea	Prosciutto	Gas flow: 5 L/min	ND	Up to 2.5 log CFU/g
			Treatment time: 0–9 min		
Lee et al. (2023)	South Korea	Sliced ham	Treatment time: 5–9 min	ATCC 19111	∼1 log CFU/g
			Voltage: 8.4 kV		
			Frequency: 2.2 kHz		

*Note*: Cold plasma systems varied substantially in gas composition, gas flow, voltage, frequency, humidity, and treatment geometry; reported outcomes are therefore shown descriptively and are not directly normalized across studies.

Song et al. (2009) evaluated different power settings (75–150 W) and treatment times (1–2 min) on inactivation of *L. monocytogenes* in sliced ham. All treatments significantly reduced *L. monocytogenes* compared with the untreated control, with higher power settings achieving greater inactivation than lower power. Inactivation was time‐dependent across all power levels, with reductions after 120 s ranging from 0.25 to 1.73 log CFU/g. Lis et al. (2018) used different air humidity (45%–90%), input voltage (6.4 and 10 kV), and frequency (2 and 10 kHz) to investigate the effect of cold plasma on *L. monocytogenes* inactivation in ham. The findings demonstrated that increasing the plasma source power significantly *enhanced L. monocytogenes* inactivation on ham surfaces. High‐power modes yielded greater reductions compared with low‐power modes. Specifically, after 20 min of treatment, *L. monocytogenes* reductions increased significantly from 0.46 log under low‐power mode to 0.91 log under high‐power mode. Furthermore, the dry low‐power mode achieved significantly greater *L. monocytogenes* inactivation compared with the wet low‐power mode. This difference may be attributed to reductions in ozone concentration under higher humidity conditions, as previously reported. Moisture‐dependent quenching of ozone by water molecules in the surrounding air or on the ham surface could limit the availability of reactive plasma species for microbial inactivation. Additionally, a thin moisture layer surrounding bacterial cells may provide a protective barrier against plasma‐generated oxidants (Lis et al. 2018; Winter et al. 2013).

Calvo et al. (2020) examined chorizo and salami at exposure times of 4, 8, and 15 min. Reductions on chorizo increased from 0.37 to 1.04 log, and on salami from 0.32 to 0.80 log. To understand the effect of cold plasma on *L. monocytogenes* inactivation on pastirma, Gök et al. (2019) evaluated oxygen, argon, and their mixtures at exposure times of 3–5 min. Treated samples showed reductions of 0.32–0.83 log CFU/cm^2^, with oxygen‐based plasmas producing greater inactivation than argon‐based plasmas. Gas composition also significantly influenced *L. monocytogenes* inactivation, with oxygen‐based plasmas achieving greater reductions than argon‐based plasmas. Lee et al. (2011) also investigated the effect of different gases (He, He + O_2_, N_2_, or N_2_ + O_2_) on the inactivation of *L. monocytogenes* on slices of cooked chicken breast and ham. *L. monocytogenes* was reduced by 1.37–4.73 log on chicken breast and by 1.94–6.52 log on ham. Gas mixtures containing oxygen produced greater inactivation than single gases, with N_2_ + O_2_ achieving the greatest reductions. These observations are consistent with mechanistic studies showing that cold plasma performance depends strongly on the reactive environment generated during treatment and on how effectively reactive species interact with the microbial cell surface and the product surface microenvironment (Olatunde et al. 2019; Qian et al. 2022).

Cold plasma produced variable inactivation of *L. monocytogenes* in RTE meats, with reductions ranging from <1 log to as high as 4 log depending on product type, treatment power, gas composition, humidity, and exposure time. Higher power settings, longer exposure time, and oxygen‐containing gas mixtures generally improved lethality, whereas moisture could reduce treatment effectiveness by quenching reactive species and altering the local surface environment. Across products, many treatments still produced only modest reductions, indicating that cold plasma performance is strongly parameter‐dependent and sensitive to matrix and surface conditions. The reviewed evidence supports cold plasma as a promising but less standardized intervention than thermal treatments, HPP, or ionizing radiation in the current RTE meat literature. In addition to this, scale‐up of cold plasma remains a major challenge, as achieving uniform plasma distribution (Sasikumar et al. 2025; Singh and Thakur 2024).

### 3.4 Comparative Performance of Thermal and Nonthermal Interventions for *L. monocytogenes* Control in RTE Meats

**FIGURE 2:**
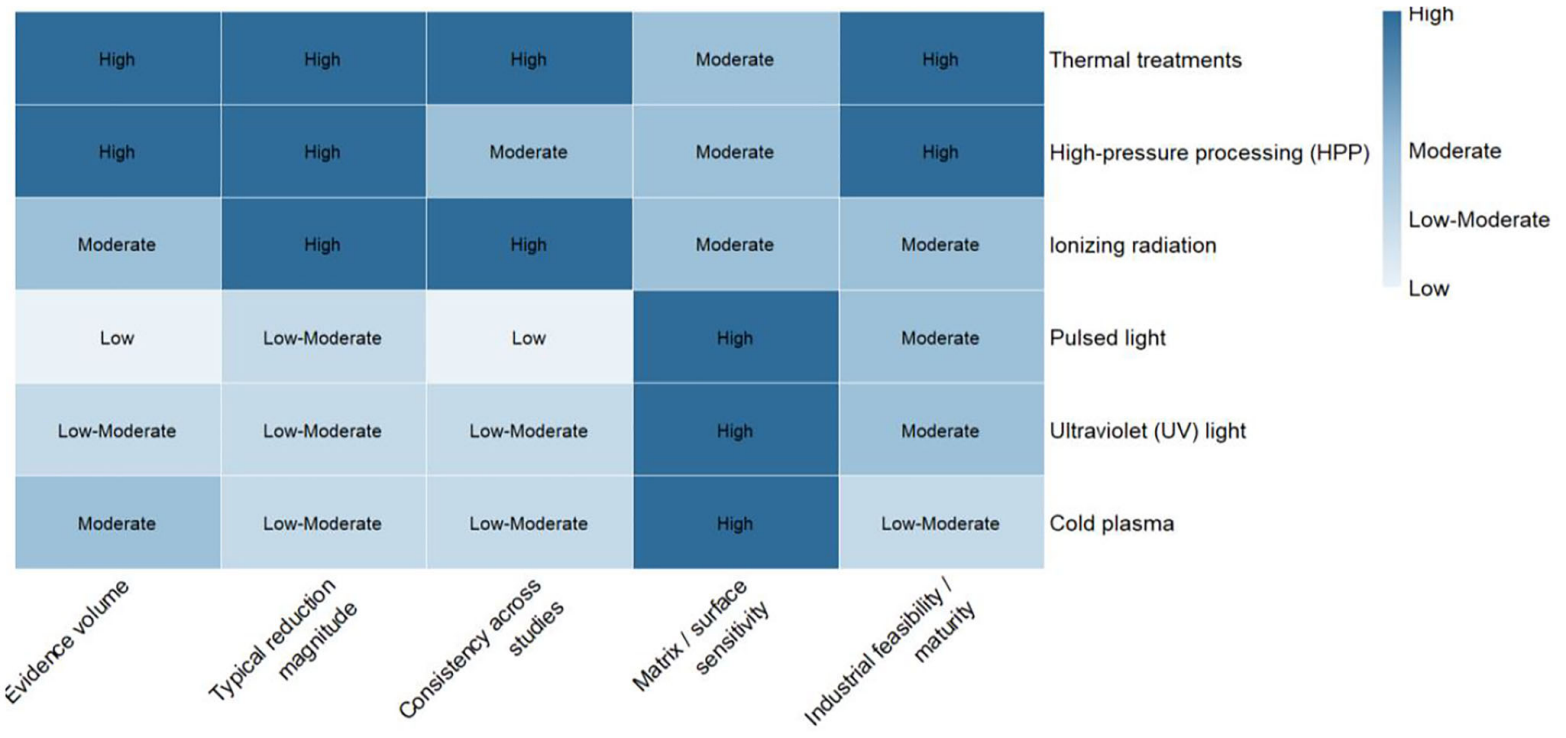
Comparison of intervention methods.

To support structured cross‐technology interpretation, the intervention classes were compared using five qualitative synthesis dimensions: evidence volume within the review, typical reported reduction range, consistency across studies, matrix or surface sensitivity, and industrial feasibility (Figure 2; Table 8). Across the intervention classes reviewed, thermal treatments showed the most consistent and predictable performance of *L. monocytogenes* in RTE meats. Their performance was governed primarily by time‐temperature intensity, and although matrix effects were evident, they were generally secondary to treatment severity. Among the nonthermal approaches, ionizing radiation and HPP produced the most substantial reductions overall, but their performance still depended on dose or pressure level, product characteristics, and the interpretation of post‐treatment behavior during storage. Ionizing radiation clusters in the high‐reduction, high‐consistency category, with most studies reporting up to 8‐log reductions at doses between 0.2 and 5.4 kGy. On the other hand, HPP occupies an intermediate but well‐defined position, characterized by high potential reduction (up to ∼7 log) but greater variability across studies, largely driven by pressure level and product composition. HPP shifts from low to high effectiveness depending on whether treatments are applied below or above ∼400 MPa (Table 3).

**TABLE 8:** Comparison of thermal and nonthermal interventions evaluated for *Listeria monocytogenes* control in RTE meats

Intervention method	Number of articles	Typical reported reduction range	Consistency across studies	Matrix/surface sensitivity	Industrial feasibility/maturity	Key interpretive note
Thermal treatments	18	Commonly multi‐log; conventional cooking and steam‐based pasteurization frequently produced the largest reductions	High	Moderate; matrix effects were present but generally secondary to time‐temperature intensity	High; already established in RTE meat processing	Thermal treatments were the most consistently effective intervention class in the reviewed literature
High‐pressure processing (HPP)	29	Minimal change to approximately 7 log, depending on pressure level and product context	Moderate	Moderate to high; influenced by fat content, product structure, strain background, and storage conditions	High to moderate; commercially established but constrained by equipment cost and product‐quality considerations	HPP showed strong nonthermal potential, but performance was highly dependent on treatment intensity and product composition
Ionizing radiation	9	All included studies reported >1‐log reductions; many reported multi‐log reductions	High	Moderate; influenced by dose, matrix structure, and storage behavior	Moderate; technically strong but constrained by public acceptance and implementation considerations	Ionizing radiation was among the most reliable nonthermal approaches in terms of substantial reduction across products
Pulsed light	4	Approximately 0.4 to 2.3 log	Low to moderate	High; strongly affected by surface properties, attachment time, and fluence	Moderate to low; attractive for surface treatment, but limited by penetration and variable efficacy	Pulsed light produced modest and condition‐dependent reductions in the reviewed RTE meat studies
Ultraviolet (UV) light	6	Approximately 0.5 to 2.0 log	Low to moderate	High; strongly constrained by topography, composition, and shielding	Moderate; easy to implement, but limited by low penetration depth and quality concerns at higher doses	UV provided measurable but generally modest reductions and functioned primarily as a surface treatment
Cold plasma	8	Less than 1 log to several logs, depending on process conditions and product type	Low to moderate	High; strongly dependent on gas composition, humidity, exposure time, and local surface conditions	Moderate to low; promising but less standardized and still challenged by scale‐up	Cold plasma showed promising but variable performance, with efficacy strongly dependent on process configuration
Cross‐cutting observation	74	Not directly comparable across all study designs	Variable	Variable	Variable	Direct comparison across technologies remained constrained by uneven evidence volume, incomplete product characterization, single‐strain designs, inconsistent reporting of variability, and limited detail on recovery conditions

In contrast, surface‐oriented technologies such as UV light and pulsed light cluster in the lower reduction range (<2 log in most studies) and show higher sensitivity to product topography and exposure conditions. Cold plasma occupies a broader range, with reductions from <1 to ∼4 log, but with high variability linked to gas composition, humidity, and treatment configuration. These technologies, therefore, exhibit lower consistency across studies and greater dependence on localized surface conditions.

### 3.5 Sources of Heterogeneity

Several recurring methodological limitations also affect interpretation across the reviewed literature. First, many studies relied on culture‐based enumeration after treatment, and apparent lethality may be influenced by the recovery conditions used. This approach may underestimate survival when stressed or injured cells fail to recover efficiently on the media applied, and therefore may overstate apparent lethality for interventions that induce sublethal rather than immediately irreversible damage. Recent work has shown that stresses in food processing can generate heterogeneous *L. monocytogenes* populations comprising healthy, sublethally injured, dormant, and dead cells, and that low‐starting concentrations together with outgrowth heterogeneity may hinder efficient detection of viable cells (Arvaniti et al. 2025). Metabolically active cells did not always recover on TSAYE or TSBYE after stress exposure, which shows that culturability alone may not fully reflect the viable fraction under some conditions (Arvaniti et al. 2025). This concern is consistent with standard detection workflows. These workflows retain enrichment steps to improve recovery efficiency, especially when cells are highly stressed or competing microbiota are present. Some protocols also include an initial recovery step before selective enrichment to resuscitate stressed organisms (D'Ambrosio et al. 2026). In addition, strain competition during selective enrichment may bias recoverability and reduce the detectability of some *L. monocytogenes* strains (EFSA Panel on Biological Hazards (BIOHAZ) 2018). For that reason, immediate reductions reported after treatment should be interpreted cautiously when recovery protocols are not described in sufficient detail.

Second, strain diversity was often limited, with many challenge studies using only a single *L. monocytogenes* strain. This limits interpretation because stress tolerance may vary with lineage background, persistence‐associated behavior, biofilm formation, and physiological adaptation to osmotic or other food‐relevant stresses. Persistent *L. monocytogenes* strains have been defined as clones repeatedly isolated from the same source or niche over time, and their persistence in food‐production environments has been linked in part to biofilm formation and enhanced survival under environmental stress and sanitation pressure (Osek et al. 2022). The available literature also indicates that biofilm‐forming ability depends on strain, origin, and environmental conditions, and that no single marker fully explains persistence across all strains and settings (Byun and Kim 2023; Osek et al. 2022). Osmotic adaptation is also relevant in meat systems. *L. monocytogenes* uses compatible‐solute transport systems involving glycine betaine and carnitine, and these osmoprotective mechanisms support survival under salt and other food‐associated stresses (Wiktorczyk‐Kapischke et al. 2021). However, the available dataset in the present review was not sufficiently standardized to support a systematic comparison across all of these strain‐level categories. These sources of heterogeneity do not invalidate the reviewed literature, but they reduce direct comparability across intervention types and highlight priorities for future challenge‐study design and reporting.

## 4 Conclusion

This scoping review mapped and synthesized the available evidence on the effectiveness of various thermal and nonthermal technologies against *L. monocytogenes* in RTE meats. Thermal treatments and ionizing radiation technologies (gamma irradiation, electron beam, and X‐ray) generally produced the most consistent and substantial reductions across the reviewed studies. In contrast, nonthermal interventions such as HPP, cold plasma, pulsed light, and UV light showed more variable performance. Regulatory benchmarks remain relevant for practical interpretation, but they should not be treated as the sole basis for assessing intervention performance. In the present review, intervention outcomes were therefore interpreted primarily in relation to treatment intensity, product conditions, matrix effects, and the distinction between immediate reduction and subsequent recovery or growth during storage.

Across studies, process efficacy was strongly influenced by treatment parameters and product characteristics. For HPP and ionizing radiation, dose and pressure levels were the main drivers of lethality, while matrix properties such as fat content and product structure modulated but did not override these effects. For light and plasma‐based technologies, surface topography, humidity, gas composition, and attachment time played critical roles and often limited inactivation to modest levels. Taken together, these patterns indicate that thermal treatments remained the most predictable intervention class, while ionizing radiation and HPP represented the most effective nonthermal approaches within the available literature. By contrast, UV, pulsed light, and cold plasma appeared more sensitive to surface conditions and process configuration, which constrained their consistency across products. These patterns suggested that thermal processes and ionizing radiation provided the most robust standalone lethality, whereas other nonthermal methods might be better suited within multistep preservation strategies. This distinction is important because industrial control of *L. monocytogenes* in RTE meats rarely depends on a single intervention in isolation, but instead reflects the combined influence of formulation, packaging, refrigeration, sanitation, environmental control, and postlethality handling.

Interpretation of the evidence was also constrained by recurring methodological limitations. Some studies relied on a single *L. monocytogenes* strain, did not report measures of variability, or expressed outcomes only as D‐values. Key product parameters, including fat content, water activity, nitrite level, and sample thickness, were frequently missed, and these gaps prevented meta‐analysis and hindered cross‐study comparison. In addition, selective enumeration alone may underestimate survival when injured cells fail to recover on selective media, and this may lead to overestimation of apparent lethality in some treatment studies. Standardized reporting, multistrain challenge designs, and systematic variation of matrix properties should be prioritized to better capture how product formulation interacts with process lethality. In addition to microbial lethality, organoleptic properties such as texture, flavor, and color must be considered, especially for nonthermal interventions. Consumer acceptance and treatment cost also play critical roles in determining the practicality and adoption of these technologies in commercial settings. These important factors were beyond the scope of this review, but remain crucial for future implementation.

Future studies should prioritize four areas. First, study design and reporting should be strengthened through multiple strain inoculation strategies, clearer reporting of variability, and more complete characterization of product composition and sample geometry. Second, greater attention is needed to injured cell recovery and to the distinction between immediate posttreatment reduction and later recovery or regrowth during storage. Third, emerging nonthermal technologies would benefit from more systematic evaluation of matrix effects, scale‐up constraints, and product quality tradeoffs under commercially relevant conditions. Finally, broader economic and implementation data are needed to clarify where these interventions are most practical within real RTE meat processing systems. Overall, the reviewed literature supports a tiered interpretation: thermal treatments remained the most consistent intervention class, HPP and ionizing radiation showed strong nonthermal potential under appropriate conditions, and surface‐oriented technologies such as UV, pulsed light, and cold plasma should be interpreted more cautiously because their performance was more context‐dependent.

## Author Contributions

**Mustafa Guzel**: conceptualization, investigation, writing – original draft, methodology, writing – review and editing. **Ilhami Okur**: conceptualization, investigation, writing – original draft, methodology, writing – review and editing, project administration, formal analysis, supervision, resources.

## Conflicts of Interest

The authors declare no conflicts of interest.
